# Postoperative blood glucose increase is associated with futile recanalization in patients with successful thrombectomy: a retrospective study

**DOI:** 10.1186/s12883-023-03512-z

**Published:** 2023-12-19

**Authors:** Tao Tang, Di Li, Tie-Ping Fan, Cong-Jie Bi, Aline M. Thomas, Man-Hong Zhao, Shen Li

**Affiliations:** 1 Department of Neurology and Psychiatry, Beijing Shijitan Hospital, Capital Medical University, No. 10 Tieyi Road, Beijing, 100038 China; 2 Department of Neurointervention, Central Hospital of Dalian University of Technology, Dalian, China; 3 Department of Anesthesiology, Central Hospital of Dalian University of Technology, Dalian, China; 4 The Russell H. Morgan Department of Radiology and Radiological Sciences, The Johns Hopkins University School of Medicine, Baltimore, MD USA; 5 Beijing Institute of Brain Disorders, Capital Medical University, Beijing, China

**Keywords:** Blood glucose increase, Futile recanalization, Acute ischemic stroke, Mechanical thrombectomy, Indicator

## Abstract

**Background:**

Timely recognition of futile recanalization might enable a prompt response and an improved outcome in post-thrombectomy patients. This study aims to evaluate whether postoperative blood glucose increase (BGI) could act as an indicator of futile recanalization in patients receiving a successful thrombectomy.

**Methods:**

This is a single-center, retrospective analysis of patients with anterior circulation large-vessel occlusion and successful thrombectomy between February 2019 and June 2022. BGI was defined as a higher level of blood glucose at the first postoperative morning than at admission. Futile recanalization was defined as patients with a modified Rankin Scale score of 3–6 at 90 days after onset. Multivariable binary logistic regression was used to assess the association of BGI with futile recanalization.

**Results:**

A total of 276 patients were enrolled, amongst which 120 patients (43.5%) had BGI. Futile recanalization was more prevalent among patients with BGI compared to those without (70.0 vs. 49.4%, *P* = 0.001). After adjusting for potential confounders, BGI was associated with a higher likelihood of futile recanalization (adjusted OR: 2.97, 95%CI: 1.50–5.86, *P* = 0.002). This association was consistently observed regardless of diabetes history, occlusion site, time from symptom onset to groin puncture, or reperfusion status.

**Conclusion:**

Our findings support BGI serving as an indicator of futile recanalization in patients with anterior circulation large-vessel occlusion and successful thrombectomy.

## Introduction

Mechanical thrombectomy has now emerged as the standard treatment for acute ischemic stroke due to proximal intracranial large-vessel occlusion [1]. However, approximately half of treated patients do not achieve functional independence despite receiving a successful recanalization [2]. This phenomenon has been termed futile recanalization [3]. As timely recognition of futile recanalization could enable a prompt response and an improved outcome, identification of its indicators has been hotly investigated in recent years [4].

Elevated blood glucose is commonly observed in patients with acute stroke [5]. The mechanisms may be due to underlying diabetes mellitus and/or stress-induced release of norepinephrine and cortisol [6]. Elevated blood glucose at admission has been reported to be associated with poor functional outcome after mechanical thrombectomy [7]. However, the relationship between elevated blood glucose at admission and futile recanalization is uncertain. Several studies found higher blood glucose at admission significantly corresponded with the likelihood of futile recanalization [8, 9], and yet other studies did not confirm the relation between elevated blood glucose at admission and futile recanalization [10, 11]. These results suggest that one isolated glucose test at admission might be insufficient for predicting functional outcome in patients with successful thrombectomy. Recently, perioperative glucose dynamics were shown to be a valuable marker for adverse outcome in patients who underwent thrombectomy [12]. Persistent ischemia during futile recanalization would theoretically induce a longer stress response that may result in a higher postoperative blood glucose compared to blood glucose at admission [5, 13]. Therefore, perioperative glucose dynamics might be a potential indicator for futile recanalization. This study aims to evaluate whether postoperative blood glucose increase (BGI) could act as an indicator of futile recanalization in patients with successful thrombectomy.

## Methods

### Study participants

Between February 2019 and June 2022, patients undergoing mechanical thrombectomy at Central Hospital of Dalian University of Technology for acute large-vessel occlusion were recruited for this retrospective study. The Central Hospital of Dalian University of Technology Ethics Committee approved the study (2019–004–11) to obtain retrospective anonymized patient data from the clinical database of the Central Hospital of Dalian University of Technology with a waiver of written informed consent. Patients were included if they (1) had a proximal anterior circulation occlusion (intracranial internal carotid artery, middle cerebral artery (M1 or M2 segment), or both); (2) were older than 18 years; (3) had a pre-stroke modified Rankin Scale (mRS) ≤ 2; (4) had a successful recanalization defined as a final modified Thrombolysis in Cerebral Infarction (mTICI) score of 2b or 3; (5) had blood tests for glucose at admission and at the first postoperative morning; and (6) had a functional outcome assessment using mRS at 90 days. The patients were managed according to current guidelines.

### Data collection and variable definitions

The following data was collected: age, sex, body mass index, pre-stroke mRS scores, medical history (hypertension, diabetes mellitus, antidiabetic treatment, previous ischemic stroke or transient ischemic attack, atrial fibrillation, and current smoking), systolic and diastolic blood pressure at admission, baseline National Institutes of Health Stroke Scale (NIHSS) score, baseline Alberta Stroke Program Early CT Score (ASPECTS), treatment with intravenous thrombolysis, occlusion site determined by digital subtraction angiography, collateral status, anesthesia type, time from symptom onset to groin puncture, time from stroke onset to reperfusion, device-pass number, reperfusion status, stroke subtype according to the Trial of Org 10172 in Acute Stroke Treatment classification [14], symptomatic intracranial hemorrhage (SICH), and blood tests (glucose, neutrophil count, and lymphocyte count) at admission and at the first postoperative morning.

Postoperative blood glucose change (BGC) was defined as the blood glucose at the first postoperative morning minus that at admission, and BGI as a BGC > 0 mmol/L. The neutrophil-to-lymphocyte ratio was calculated by dividing the neutrophil count by the lymphocyte count. Collateral status was evaluated at the pre-thrombectomy angiogram, which was dichotomized into good (grade 3–4) and poor (grade 0–2) collaterals according to the American Society of Interventional and Therapeutic Neuroradiology/Society of Interventional Radiology collateral flow grading system [15]. Reperfusion status was evaluated using the mTICI score [16]. SICH was defined as evidence of intracranial hemorrhage associated with an increase of 4 or more points on the NIHSS scores within 24 h after onset [17]. Imaging variables were analyzed by two experienced neurointerventionalists (> 10 years of experience) blinded to patient information.

### Outcome

Futile recanalization was defined as patients with a mRS score of 3–6 at 90 days after onset, which was assessed by stroke neurologists during the clinical follow-up visits or via standardized telephone interviews with the patients or their caregivers.

### Statistical analysis

Shapiro-Wilk test was used to test data distribution. Categorical variables were expressed as frequencies and percentages. Continuous variables were expressed as mean ± standard deviation (SD), or median (interquartile range (IQR)) when non-normally distributed. Baseline characteristics were compared using Student t test/Mann–Whitney U test, or χ2 test/Fisher’s exact test, as appropriate according to the type of variables and their distribution.

A restricted cubic spline model was performed to examine the shape of the correlation between BGC and futile recanalization. We selected four knots at 5th, 35th, 65th and 95th. The associations between BGC or BGI and futile recanalization was then evaluated by multivariable logistic regression models, adjusted for potential confounders (age, diastolic blood pressure at admission, baseline NIHSS score, blood glucose levels at admission, occlusion site, collateral status, time from stroke onset to reperfusion, and SICH). These confounders were selected based on statistical significance (*P* < 0.05) in the univariable analysis and the potential to affect patient outcomes [18]. Multiplicative interaction analyses were performed to evaluate the heterogeneity of the association of postoperative BGI with futile recanalization between subgroups of different categories including diabetes history, occlusion site (internal carotid artery vs. middle cerebral artery), time from symptom onset to groin puncture (≤ 360 vs. > 360 min), and reperfusion status (mTICI 2b vs. mTICI 3). Finally, we performed a sensitivity analysis on the likelihood of futile recanalization by comparing normoglycemic patients with BGI (≤ 7.8 mmol/L at the first postoperative morning) to those without BGI. We reported adjusted odds ratios (aOR) for multivariable analyses with a 95% confidence interval (CI). All tests were 2-tailed with a significance level of 0.05. All analyses were performed with STATA software version 17 (StataCorp LLC) and R software version 4.2.3.

## Results

### Baseline characteristics

**Fig. 1:**
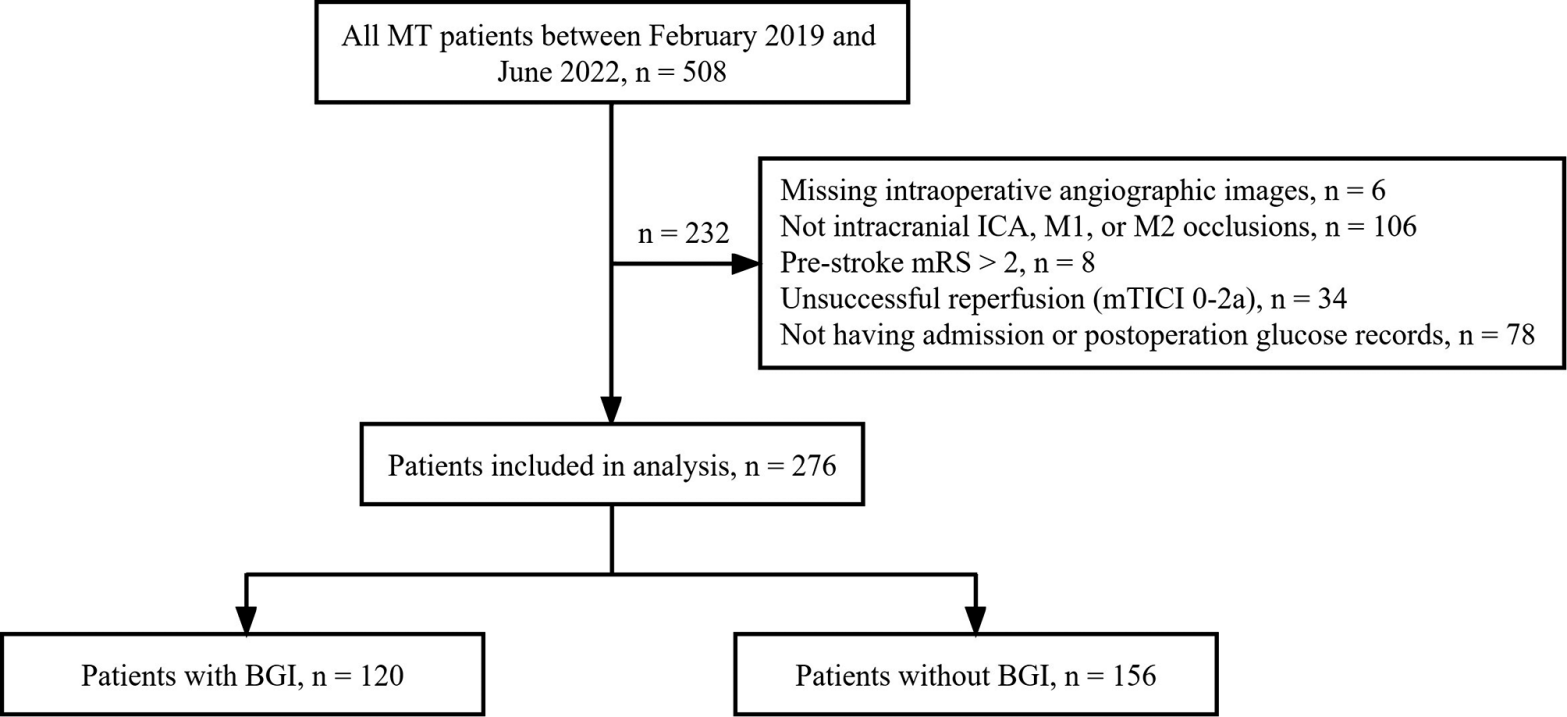
Flowchart illustrating the study inclusion/exclusion and grouping process. *MT* mechanical thrombectomy, *ICA* internal carotid artery, *M1, M2* the first and second segment of middle cerebral artery, *mRS* modified Rankin Scale, *mTICI* modified Thrombolysis in Cerebral Infarction, *BGI* blood glucose increase

**Fig. 2:**
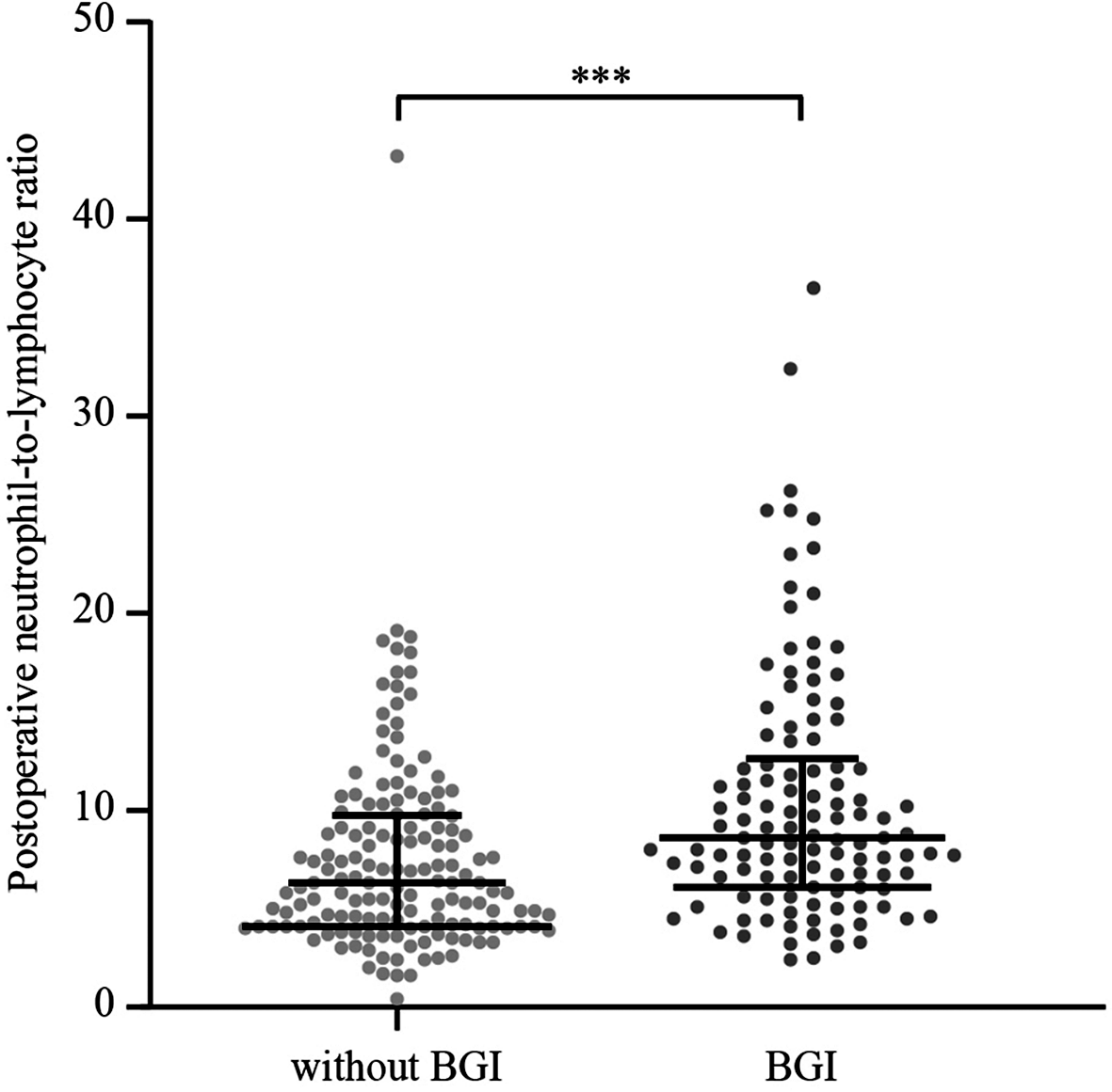
The postoperative neutrophil-to-lymphocyte ratio was higher in patients with BGI than those without (8.6 [6.1–12.3] vs. 6.3 [4.1–9.7], *P* < 0.001). *BGI* blood glucose increase

**Fig. 3:**
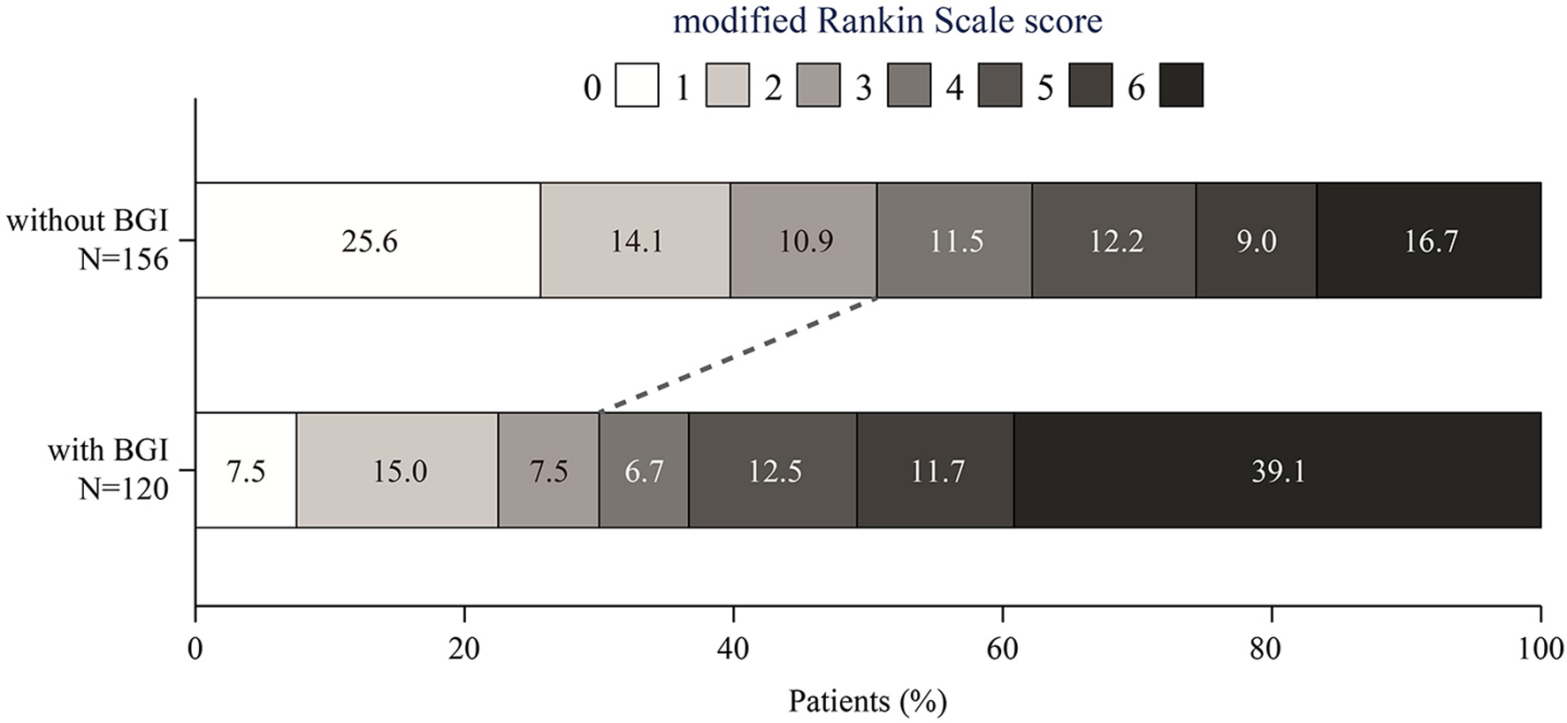
Distribution of modified Rankin Scale scores at 90 days after stroke according to postoperative BGI. *BGI* blood glucose increase

The patient selection process is illustrated in Fig. 1. A total of 276 patients were included in this study. The median age was 69 (62–77) years and 88 patients (31.9%) were female. The patients had a median NIHSS score of 17 (13–22). Table 1 summarizes the baseline characteristics of all patients and patients with/without BGI. In total, 120 patients (43.5%) had BGI. Compared to patients without BGI, those with BGI had lower diastolic blood pressure (77.3 vs. 82.4 mmHg, *P* = 0.005) and blood glucose at admission (6.9 vs. 7.6 mmol/L, *P* = 0.001), as well as higher NIHSS scores (18 vs. 16, *P* = 0.009) and longer onset to reperfusion time (334 vs. 307 min, *P* = 0.033). The rates of SICH were similar in patients with and without BGI (6.7% vs. 9.0%, *P* = 0.483). The postoperative neutrophil-to-lymphocyte ratio was higher in patients with BGI than those without (8.6 [6.1–12.3] vs. 6.3 [4.1–9.7], *P* < 0.001; Fig. 2). Futile recanalization was more prevalent among patients with BGI compared to those without (70.0 vs. 49.4%, *P* = 0.001; Fig. 3).

**Table 1:** Baseline characteristics of all patients and patients with/without BGI

Variables	All patients (n = 276)	Patients with BGI (n = 120)	Patients without BGI (n = 156)	*P* value
Age, years, median (IQR)	69 (62–77)	71 (62–79)	69 (62–75)	0.067
Sex, female, n (%)	88 (31.9)	36 (30.0)	52 (33.3)	0.556
BMI, kg/m^2^, mean ± SD	24.6 ± 3.0	24.7 ± 3.4	24.6 ± 2.7	0.775
Pre-stroke mRS ≥ 1, n (%)	16 (5.8)	4 (3.3)	12 (7.7)	0.124
**Medical history, n (%)**				
Hypertension	148 (53.6)	66 (55.0)	82 (52.6)	0.687
Diabetes mellitus	63 (22.8)	29 (24.2)	34 (21.8)	0.642
Antidiabetic treatment	38 (13.8)	18 (15.0)	20 (12.8)	0.602
Ischemic stroke/TIA	32 (11.6)	13 (10.8)	19 (12.2)	0.729
Atrial fibrillation	115 (41.7)	54 (45.0)	61 (39.1)	0.325
Current smoking	113 (40.9)	47 (39.2)	66 (42.3)	0.599
**Current stroke event**				
SBP, mmHg, mean ± SD	144.2 ± 26.9	140.9 ± 28.3	146.7 ± 25.6	0.080
DBP, mmHg, mean ± SD	80.2 ± 15.4	77.3 ± 15.3	82.4 ± 15.2	0.005
Baseline NIHSS score, median (IQR)	17 (13–22)	18 (14–25)	16 (12–21)	0.009
ASPECTS, median (IQR)	8 (7–10)	8 (7–10)	8 (7–10)	0.586
Blood glucose, mmol/L, median (IQR)	7.3 (6.4–9.5)	6.9 (6.3–8.2)	7.6 (6.6–10.1)	0.001
Intravenous thrombolysis, n (%)	135 (48.9)	53 (44.2)	82 (52.6)	0.167
Occlusion site, n (%)				0.172
M1	133 (48.2)	51 (42.5)	82 (52.5)	
M2	27 (9.8)	11 (9.2)	16 (10.3)	
Intracranial ICA	116 (42.0)	58 (48.3)	58 (37.2)	
Poor collaterals, n (%)	74 (26.8)	29 (24.2)	45 (28.8)	0.384
Anesthesia, n (%)				0.394
General anesthesia	12 (4.4)	6 (5.0)	6 (3.9)	
Local anesthesia	137 (49.6)	54 (45.0)	83 (53.2)	
Conscious sedation	127 (46.0)	60 (50.0)	67 (42.9)	
OPT, min, median (IQR)	260 (180–355)	278 (185–395)	246 (178–336)	0.049
ORT, min, median (IQR)	320 (241–415)	334 (256–453)	307 (240–393)	0.033
Device-pass number, median (IQR)	2 (1–3)	2 (1–3)	1 (1–2)	0.135
Reperfusion status, n (%)				0.296
mTICI 2b	135 (48.9)	63 (52.5)	72 (46.2)	
mTICI 3	141 (51.1)	57 (47.5)	84 (53.8)	
Stroke subtype, n (%)				0.698
Cardioembolism	150 (54.4)	63 (52.5)	87 (55.8)	
Large-artery atherosclerosis	119 (43.1)	53 (44.2)	66 (42.3)	
Others	7 (2.5)	4 (3.3)	3 (1.9)	
SICH	22 (8.0)	8 (6.7)	14 (9.0)	0.483
Futile recanalization, n (%)	161 (58.3)	84 (70.0)	77 (49.4)	0.001

*BGI* blood glucose increase, *BMI* body mass index, *mRS* modified Rankin Scale, *TIA* transient ischemic attack, *SBP* systolic blood pressure, *DBP* diastolic blood pressure, *NIHSS* National Institutes of Health Stroke Scale, *ASPECTS* Alberta Stroke Program Early CT Score, *M1, M2* the first and second segment of middle cerebral artery, *ICA* internal carotid artery, *OPT* onset to groin puncture time, *ORT* onset to reperfusion time, *mTICI* modified Thrombolysis in Cerebral Infarction, *SICH* symptomatic intracranial hemorrhage

### Relationship between BGC/BGI and outcome

**Fig. 4:**
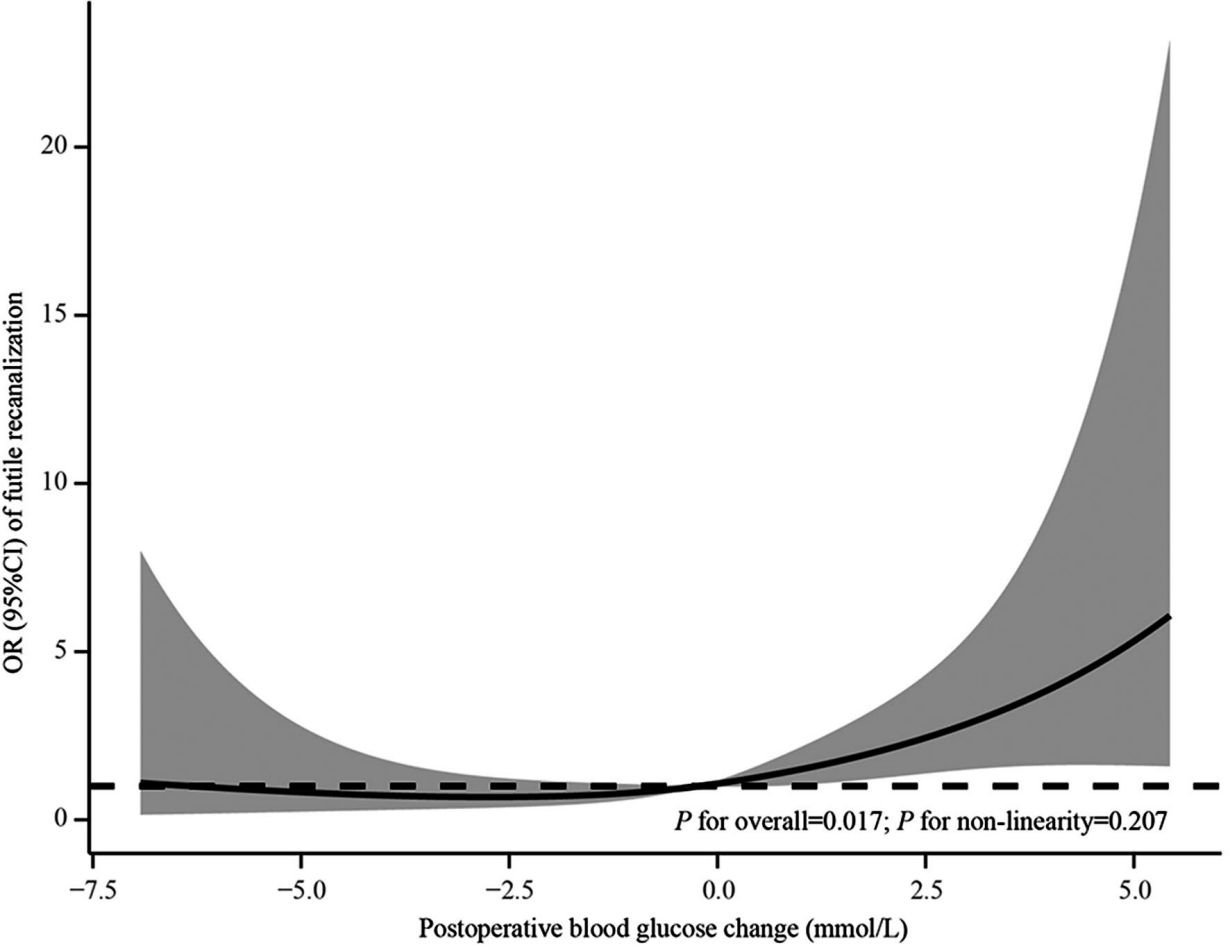
Cubic model of the association between postoperative blood glucose change and futile recanalization after adjusting for age, diastolic blood pressure at admission, baseline National Institutes of Health Stroke Scale score, blood glucose levels at admission, occlusion site, collateral status, time from stroke onset to reperfusion, and symptomatic intracranial hemorrhage

**Fig. 5:**
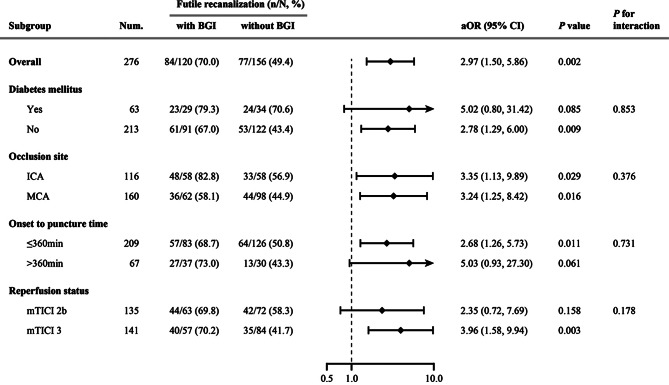
Influence of BGI on futile recanalization in all patients and according to diabetes history, occlusion site, time from symptom onset to groin puncture, and reperfusion status. aOR was adjusted for age, diastolic blood pressure at admission, baseline NIHSS score, blood glucose levels at admission, occlusion site, collateral status, time from stroke onset to reperfusion, and SICH. *BGI* blood glucose increase, *aOR* adjusted odds ratios, *NIHSS* National Institutes of Health Stroke Scale, *CI* confidence interval, *ICA* internal carotid artery, *MCA* middle cerebral artery, *mTICI* modified Thrombolysis in Cerebral Infarction, *SICH* symptomatic intracranial hemorrhage

In the restricted cubic spline regression model, the correlation between postoperative BGC and futile recanalization was linear (*P* for overall = 0.017; *P* for non-linearity = 0.207; Fig. 4). In multivariable analyses, postoperative BGC, as a continuous variable, was associated with an increased risk for futile recanalization (per 1 mmol/L: aOR: 1.23, 95%CI: 1.07–1.41, *P* = 0.003). Similarly, BGI, as a categorical variable, also increased the likelihood of futile recanalization (aOR: 2.97, 95%CI: 1.50–5.86, *P* = 0.002; Table 2). In subgroup analyses, BGI was associated with futile recanalization in patients with mTICI 3 reperfusion (aOR: 3.96, 95%CI 1.58–9.94, *P* = 0.003), but not in those with mTICI 2b reperfusion (aOR: 2.35, 95%CI 0.72–7.69, *P* = 0.204). However, an insignificant interaction between reperfusion status and BGI on futile recanalization was observed in the multiplicative interaction analysis (*P* = 0.178). Heterogeneity in the association of BGI with futile recanalization was also not observed when segmented based on diabetes history, occlusion site, or time from symptom onset to groin puncture (Fig. 5). The sensitivity analysis showed that normoglycemic patients with BGI were also prone to have futile recanalization compared to those without BGI (aOR: 2.68, 95%CI: 1.08–6.67, *P* = 0.034).

**Table 2:** Logistic regression of futile recanalization in patients with successful thrombectomy

Variable	Univariate analysis		Multivariate analysis	
	OR (95% CI)	*P* value	aOR (95% CI)	*P* value
Age	1.07 (1.05, 1.10)	< 0.001	1.09 (1.05, 1.12)	< 0.001
DBP at admission	0.99 (0.98, 1.01)	0.325	1.00 (0.98,1.03)	0.675
NIHSS at admission	1.18 (1.12, 1.24)	< 0.001	1.17 (1.11, 1.25)	< 0.001
Glucose at admission	1.18 (1.07, 1.29)	0.001	1.28 (1.14, 1.44)	< 0.001
MCA occlusion	0.43 (0.26, 0.71)	0.001	0.40 (0.21, 0.76)	0.005
Good collateral status	0.54 (0.32, 0.93)	0.025	0.45 (0.23, 0.91)	0.026
Onset to reperfusion time	1.00 (1.00, 1.00)	0.177	1.00 (1.00, 1.00)	0.153
SICH	1.59 (0.62, 4.02)	0.332	1.77 (0.50, 6.32)	0.378
BGI	2.39 (1.45, 3.95)	0.001	2.97 (1.50, 5.86)	0.002

*DBP* diastolic blood pressure, *NIHSS* National Institutes of Health Stroke Scale, *MCA* middle cerebral artery, *SICH* symptomatic intracranial hemorrhage, *BGI* blood glucose increase

## Discussion

This study demonstrated an association between postoperative BGI and futile recanalization in patients with acute anterior circulation large-vessel occlusion and successful thrombectomy. This association was consistently observed regardless of diabetes history, occlusion site, time from symptom onset to groin puncture, or reperfusion status.

In this study, we evaluated the relationship between glucose dynamics and futile recanalization. Previously, the predictive value of blood glucose dynamics had been evaluated in patients treated with intravenous thrombolysis using persistent hyperglycemia (≥ 144 mg/dL at admission and 24–48 h after onset) as a metric [19, 20]. Persistent hyperglycemia in these studies was linked to poorer functional outcomes. In another study, early persistent hyperglycemia (> 140 mg/dL at admission and 24 h after thrombectomy) in patients undergoing thrombectomy predicted adverse outcomes [12]. Instead of evaluating persistent hyperglycemia with a specific value as in previous studies, we introduced a new metric, BGI, to compare the levels of blood glucose after thrombectomy and at admission, which has the potential to accommodate patient-to-patient heterogeneity in blood glucose levels. Although not adequately evaluated in previous studies, it is assumed that BGI may be relevant to futile recanalization, even in normoglycemic cases. BGI is commonly observed after stroke, though not necessarily to hyperglycemic levels (> 140 mg/dL) [5]. In this circumstance, elevated blood glucose may still reflect sustained stress responses in futile recanalization [13]. Therefore, we selected BGI for evaluating glycemic dynamics in this study.

Our results showed that BGI was more often observed in patients with futile recanalization compared with those without. Furthermore, a multivariable regression showed BGI was associated with futile recanalization. The association maintained in subgroup analyses. However, the causative relationship between BGI and futile recanalization has yet to be proven. On the one hand, it is possible that a longer stress response due to futile recanalization could induce higher postoperative glucose levels compared to those at admission [13]. On the other hand, futile recanalization is thought to result from multiple mechanisms including inflammatory responses, no-reflow phenomenon, and reperfusion injury [21–23]. BGI may reveal persistently elevated glucose levels within 24 h after onset. This could lead to harmful effects in patients with successful thrombectomy through exacerbation of inflammatory responses, impaired cerebrovascular reactivity, intracellular acidosis, and increased vulnerability to reperfusion injury [6, 24].

Glucose lowering has not been demonstrated to be beneficial for patients with stroke in clinical trials [25, 26], few of which have evaluated the benefit in the thrombectomy setting. Our result might provide insight for future trials on post-thrombectomy glucose management. First, blood glucose level at admission might be a potential reference value for individualized glucose management. As stress-induced elevated glucose may initially function to provide the metabolic fuel needed to repair ischemic brain issue [27], early glucose control based on one standard cut-off value may be iatrogenic, impairing cerebral function particularly in patients with futile recanalization. Second, reperfusion status should be taken into account for glucose control during the acute phase [28]. BGI was associated with a higher likelihood of futile recanalization in patients with mTICI 3 reperfusion, implying that illogical blood glucose increase after complete reperfusion is detrimental. In contrast, BGI was not associated with futile recanalization in mTICI 2b reperfusion, indicating that blood glucose increase after incomplete reperfusion may be a protective compensation [29]. Therefore, we suggest exercising caution when lowering glucose in patients with incomplete reperfusion.

This study has several limitations. First, it is a single-center, retrospective study, which could inevitably cause selection bias. However, the results obtained seem both pathophysiologically plausible and clinically relevant. A prospective study is warranted to confirm the results obtained from this study. Second, BGI, defined by changes of perioperative blood glucose, could be affected by multiple factors, such as feeding status and postoperative hypoglycemic treatment, which might influence its reliability. Third, we assessed the dynamics of perioperative blood glucose only by two blood glucose tests, which may induce relatively high variability. Continuous glucose monitoring might be a preferable approach for future research to further elucidate the association between the dynamics of perioperative blood glucose and clinical outcomes in patients with successful recanalization. Nevertheless, the relative ease of obtaining BGI in a real-world clinical setting might compensate for the above shortcomings.

## Conclusions

Our findings support postoperative BGI serving as an independent indicator of futile recanalization in patients with anterior circulation large-vessel occlusion and successful thrombectomy.

## Data Availability

The data are available from the corresponding author (Shen Li, Email: lishen@mail.ccmu.edu.cn) upon reasonable request.

## Abbreviations

BGI: Blood glucose increase
M1, M2: The first and second segment of middle cerebral artery
mRS: Modified Rankin Scale
mTICI: Modified Thrombolysis in Cerebral Infarction
NIHSS: National Institutes of Health Stroke Scale
ASPECTS: Alberta Stroke Program Early CT Score
SICH: Symptomatic intracranial hemorrhage
BGC: Blood glucose change
SD: Standard deviation
IQR: Interquartile range
aOR: Adjusted odds ratios
CI: Confidence interval
